# A Retrospective Outbreak Investigation of a COVID-19 Case Cluster in a Berlin Kindergarten, November 2020

**DOI:** 10.3390/ijerph19010036

**Published:** 2021-12-21

**Authors:** Sebastian Ruf, Franziska Hommes, Welmoed van Loon, Joachim Seybold, Tobias Kurth, Marcus A. Mall, Frank P. Mockenhaupt, Stefanie Theuring

**Affiliations:** 1 Institute of Tropical Medicine and International Health, Charité—Universitätsmedizin Berlin, Corporate Member of Freie Universität Berlin and Humboldt Universität zu Berlin, 13353 Berlin, Germany; sebastian.ruf@charite.de (S.R.); franziska.hommes@charite.de (F.H.); welmoed.van-loon@charite.de (W.v.L.); frank.mockenhaupt@charite.de (F.P.M.); 2 Medical Directorate, Charité—Universitätsmedizin Berlin, Corporate Member of Freie Universität Berlin and Humboldt Universität zu Berlin, 10117 Berlin, Germany; joachim.seybold@charite.de; 3 Institute of Public Health, Charité—Universitätsmedizin Berlin, Corporate Member of Freie Universität Berlin and Humboldt Universität zu Berlin, 10117 Berlin, Germany; tobias.kurth@charite.de; 4 Department of Pediatric Respiratory Medicine, Immunology and Intensive Care Medicine, Charité—Universitätsmedizin Berlin, Corporate Member of Freie Universität Berlin and Humboldt Universität zu Berlin, 13353 Berlin, Germany; marcus.mall@charite.de

**Keywords:** COVID-19, SARS-CoV-2, kindergarten, daycare, outbreak, case clusters

## Abstract

While SARS-CoV-2 infection activity in German kindergartens during the first year of the pandemic appeared to be overall low, outbreaks did occur. We retrospectively investigated an outbreak in November and December 2020 in a Berlin kindergarten participating in the Berlin Corona School and Kindergarten Study (BECOSS). Interviews were conducted with affected families regarding symptomatology, contact persons and possible sources of infection, as well as relevant information on the conditions on-site and infection prevention measures. A chronology of the outbreak was elaborated, and based on data on contacts and symptoms, we mapped the most likely chains of infection. Overall, 24 individuals, including ten educators, seven children, and seven household members, were infected with SARS-CoV-2 in a four-week time interval. Courses of infection ranged from asymptomatic to severe, with children less affected by symptoms. Viral spread within the facility seemed to occur mainly through kindergarten staff, while children primarily transmitted infections within their families. Interviewees reported that hygiene measures were not always adhered to inside the facility. To prevent outbreaks in kindergartens, especially in the light of current and newly emerging viral variants of concern, strict compliance to hygiene rules, staff vaccinations against SARS-CoV-2, and immediate reaction to suspected cases by quarantining and frequent testing seem reasonable measures.

## 1. Introduction

In the ongoing SARS-CoV-2 pandemic, there have been disputing opinions on the role young children and kindergartens play regarding infection dynamics [1,2,3]. Children between 0–9 years are estimated to be significantly less susceptible to SARS-CoV-2 infection [4,5], and COVID-19 is usually less severe and often asymptomatic in young children, linked with significantly lower viral loads compared to symptomatic infections [6]. While young children can still effectively transmit the virus [2], evidence to date rather suggests that they are not among the main drivers of the pandemic and that kindergartens do not represent silent transmission reservoirs [1,6,7,8,9,10]. However, SARS-CoV-2 outbreaks have been reported in kindergartens in Germany and elsewhere [11,12,13].

Within the first COVID-19 wave in Germany, starting in March 2020, Berlin kindergartens fully closed or ran for emergency care during a first six weeks of lockdown. After having reopened in May 2020, they had to close again except for emergency care during the second COVID-19 wave in mid-December 2020. The new German Infection Protection Act, which came into effect as of 23 April 2021 during the third COVID-19 wave, regulates the closing of kindergartens and schools depending on the current incidence rate [14]. Since 17 May 2021, daycare centers have been returning to normal operation, with infection prevention regulations in place. Continuous attendance of kindergartens is vital for young children in terms of early education, social contacts, and developmental challenges as well as for parents in offering time free from child custody [15,16]. Despite the decision to disrupt the daily routine of young children and their families for preventive reasons, there is still surprisingly little insight in how infection clusters in kindergartens might contribute to overall community transmission. Limited data are available on infection chains and their effective handling in daycare facilities, which could become particularly important in the light of new viral variants of concern and a change in age-specific infection incidence as vaccination rates in the adult population increase, while pre-school aged children to date have no option of being vaccinated against COVID-19.

By conducting an in-depth investigation of a SARS-CoV-2 outbreak in a Berlin kindergarten at the peaking second COVID-19 wave, we aimed at contributing to the knowledge on transmission dynamics needed for informed decision making in educational settings of the youngest age group.

## 2. Materials and Methods

We conducted a qualitative retrospective outbreak investigation, describing the circumstances on site, prevention methods in place, and the most likely chains of infection.

### 2.1. Study Setting

The Berlin Corona School and Kindergarten Study (BECOSS) investigated prevalence of acute SARS-CoV-2 infections by oro-nasopharyngeal swabs (PCR) among study participants, SARS-CoV-2 antibodies, institutional infection prevention measures, as well as individual hygiene behavior and quality of life at four different timepoints since June 2020. Methods and results of the overall study have been published previously [10,17]. The study setting included 12 randomly selected Berlin kindergartens, where our first study round took place in September 2020. At that time, we did not detect any SARS-CoV-2 infection in participating children, staff, and household members [10]. In November and December 2020, one study site reported the occurrence of several confirmed SARS-CoV-2 cases to the study team. The overall seven-day-incidence of SARS-CoV-2 in children from 0–4 years in Berlin was 61.6 per 100.000 inhabitants at that time [18]. Vaccinations against SARS-CoV-2 were not yet available at that time for any age group, and the wild-type virus predominated. The governmental testing strategy did not include routine screening of asymptomatic kindergarten children at that time.

### 2.2. Data Collection and Analysis

During the outbreak between November and December 2020, 24 individuals connected with the kindergarten were diagnosed with a SARS-CoV-2 infection confirmed by RT-PCR test. From these 24 individuals, nine were tested within the BECOSS at Charité-Universitätsmedizin Berlin, and 15 tested individually at their family doctor or a public testing site. Individuals underwent testing because of symptoms, because of contact with infected persons in the kindergarten or because of an infected family member. RT-PCR confirmed infections were reported to the Berlin health authorities by the testing institution according to the German infection protection law. At that time, health authorities were largely overburdened, so there was no stringent follow-up or outbreak investigation conducted by the respective district health office. In February 2021, we approached the 24 affected individuals for retrospective in-depth interviews; 23 of them (parents in case of infected children) agreed to be interviewed. Interviews were conducted individually for every infected household member in 14/15 affected households. A semi-structured questionnaire was designed and applied, including sociodemographic information, questions about the interviewee’s role inside the daycare center, and a chronological reconstruction of relevant dates and events, followed by a description of the symptoms and an assessment of all contacts of this person within the assumed infectious interval. Participants were also asked for close contacts to any positively tested person and the most likely source of infection. Interviews were conducted either via telephone (13/14 households) or face-to-face (1/14) at Charité—Universitätsmedizin Berlin by the study team and lasted about 20 min each. An additional in-depth interview was conducted with the head of the kindergarten to obtain general data on the facility, group size, separate rooms for group division, applied infection prevention measures, and the chronology of the outbreak.

The day of symptom onset was defined as the first day when the person reported any symptoms suspicious of COVID-19. Those were fever, cough, general cold symptoms, chill, headache, chest pain, fatigue, diarrhea, nausea, emesis, stomachache, limb pain, and loss of taste and smell [11]. We defined the infectious period as two days before symptom onset until fourteen days afterwards and an average incubation period of six days [19,20].

**Figure 1:**
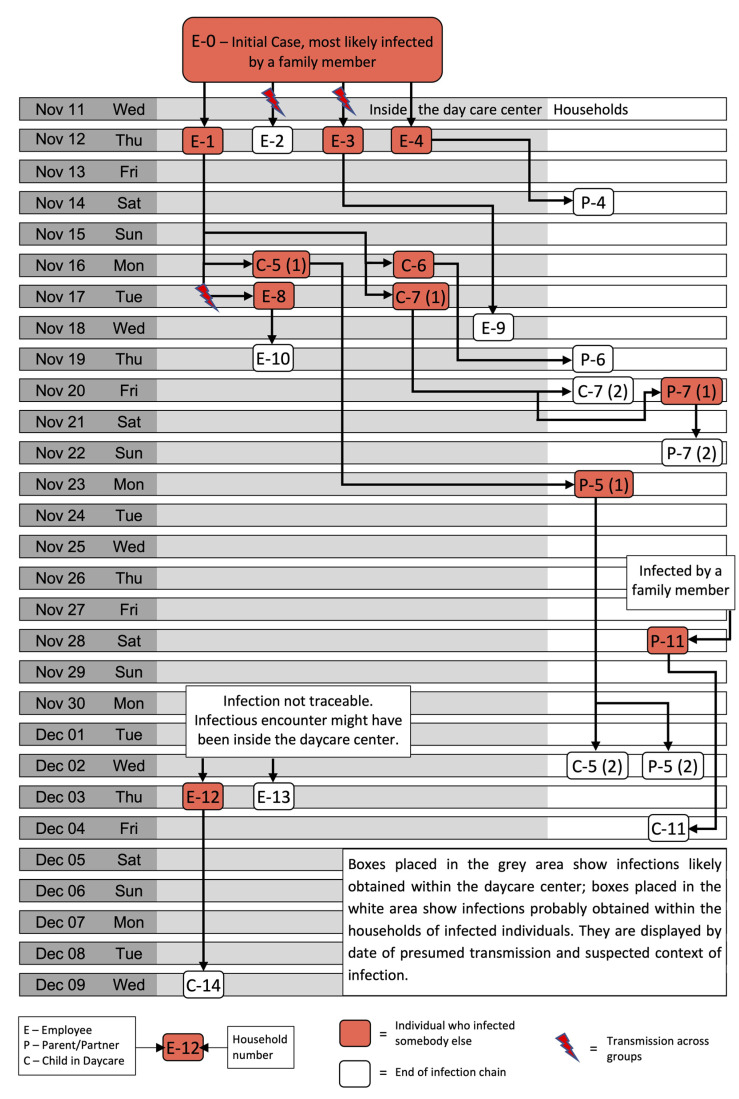
Infection chain of a COVID-19 outbreak in a kindergarten in November/December 2020 in Berlin, Germany.

For analysis, we categorized and structured findings from the interviews and elaborated an outbreak chronology. We conducted in-depth contact assessments (intra- and inter-household and across the facility), considering prevention methods reported by the interviewee (e.g., wearing facemask or keeping distance) when having contact with infected individuals. We assumed transmission within infection chains plausible within the infectious period of the index person and within the incubation period of the infected person and established the most likely day of transmission by triangulation with contact history. This information was used to graphically map the most likely chains of infection (see Figure 1).

## 3. Results

### 3.1. Institutional Hygiene Concept

The facility consisted of six groups, each with about 20 children (age range 0.5–6 years) and 2–5 educators. All groups were separated from each other for infection prevention and had different timeslots for lunch breaks except for two groups due to spatial limitations. Educators were assigned to fixed groups except for substitution in case of illness. Keeping physical distance and wearing masks was compulsory for all adults outside the group rooms. Inside the group rooms, no rule for face covering existed. Interviews revealed that only few educators used a mask within the group rooms, but the majority did not wear masks. Children were not wearing masks at any time. Parents had to wear a mask when dropping or collecting their child, and only one parent per family was allowed inside for that purpose. Hand disinfection was obligatory at the entrance and accessible in every room for parents and staff. Children were instructed to wash their hands with soap and water before entering rooms. An absence rule for symptomatic staff was not in place due to limited human resources. The kitchen staff disinfected facility surfaces, such as door handles, on a daily base; being responsible for the food supply, they had contact with all groups. Information obtained via interviews revealed that particularly group separation was implemented inconsistently since a common staff break room was accessed by all employees without any infection prevention measures.

### 3.2. Description of the Case Cluster

The 24 retraced SARS-CoV-2 infections affected five of six groups and the kitchen staff. Of the 24 individuals, ten were staff, seven children, and seven household members. The reconstruction of the most likely chains of infections is depicted in Figure 1.

The suspected index case, which was an educator of group 1, most likely was infected by a family member. This symptomatic person was the assumed origin of infections among four staff members of two different groups (group 1 and 2) and the kitchen on 12 November, which is the presumed date of transmission. Two of these staff members already developed symptoms on 14 November, and only one of them abstained from working right away. Children of the affected groups 1 and 2 were not quarantined before the first positive test result was obtained on 17 November. In addition, one of the symptomatic staff members still went to work on 16 and 17 November. This facilitated the second generation of infections: three children of group 1, two staff members of group 3 and the kitchen, and one household member (linked with group 1) were infected. Since affected groups 1 and 2 then went into quarantine on 18 November, the third and fourth generation of infections mainly included household members within group 1, of which two were siblings also visiting the facility. In the not-immediately quarantined group 3, another transmission very likely occurred between two educators on 19 November. One parent became infected on 28 November, followed by one child, both without a known connection to the outbreak inside the kindergarten based on anecdotal evidence. Two educators of group 4 and 5 tested positive in the beginning of December without any identified infectious encounter. However, we could not exclude the possibility of a connection to the outbreak. Both stated that they likely became infected during work, and they had no knowledge of a positive case within their private environment. The last reported case was a child in group 5 probably infected by one of these educators on 9 December. This sums up to thirteen cases in the first group, two in the second, four in the third, one in the fourth, two in the fifth group, and two cases among the kitchen staff.

### 3.3. Reported Symptoms

Of seven infected children, none had symptoms for longer than five days, and none had a severe course leading to a need for hospitalization. Five had fever, four had signs of a common cold, and three had gastrointestinal complaints. Another three had signs of prostration, one child was asymptomatic, and none had a loss of smell or taste.

Of the sixteen interviewed adults, seven had fever, fourteen had signs of a common cold, six had gastrointestinal complaints, twelve had signs of prostration, and ten reported a loss of taste and smell. None of the known adult cases was asymptomatic. The assessed symptom constellations per individual are displayed in Table 1 and Table 2. The duration of illness ranged from one week to longer-term symptoms, such as fatigue and chest pain, and, in one case, to subsequent severe conditions, including pericardial effusion and myocarditis.

## 4. Discussion

This outbreak investigation in a kindergarten in Berlin at an overall community incidence rate of about 210 per 100.000 inhabitants [21] contributes to the evidence on the role of these facilities in the context of infection dynamics. We observed seven cases of infected children, all showing rather mild symptoms or asymptomatic courses. This is in accordance with other studies as shown in a systematic review by Ludvigsson, 2021 [8]. An antibody screening from the German state of Bavaria concludes that 47% of children have asymptomatic courses [22]. Consequently, in this outbreak, about half the cases of child infections might have been missed. However, as contact to an infected individual often led to testing of all family members regardless of having symptoms or not, the number of undetected asymptomatic infections is likely to be low in this setting. Several studies have shown that children spread the virus among their relatives quite frequently [2,23,24]. This might be related to the fact that, for parents, it is virtually impossible to keep physical distance from an infant or young child. Through continued close contact and exposure, further enforced if a family is quarantined in a constricted living environment, virus transmission even in case of a low viral load is imaginable [2,23,25]. Notably, there was no case of an infection suggested through an asymptomatic child.

Rather surprisingly, we did not find any evidence for a case where an infected child infected another child or an educator inside the daycare center. In accordance, a large meta-analysis on the epidemiology of COVID-19 among young children suggests limited evidence of infections passed on from children to others and an overall unclear role of children in transmitting COVID-19 [24]. A surveillance of contact persons in the educational context in Frankfurt, Germany, as well as another study in southwest Germany equally showed that kindergarten children were not the drivers of infections [26,27]. Presumably, most onward transmissions in our investigated kindergarten outbreak happened through caretakers. Interviewees reported inconsistent intra-facility infection prevention and control measures; especially group separation was not effectively implemented since staff from all groups accessed the staff break room without distancing or masks. This might have contributed substantially to cross-group transmission and would have been easily preventable through more rigorous implementation of those rules.

In addition to a more thorough hygiene concept, e.g., including group-wise staggering of break times of staff, further actions on infection prevention seem advisable. With an immediate reaction to the suspected cases, for example, with an absence rule for all symptomatic individuals, this outbreak might have been terminated after the first generation of viral spread. Furthermore, a testing scheme of two rapid-tests per week for employees and children and the availability of SARS-CoV-2 vaccinations for adults and children aged at least 12 years could help to reduce infection transmission to a level allowing kindergartens to remain open. Kindergarten staff in Berlin had the opportunity to receive vaccinations since April 2021 [28], which, in the light of our findings, is a fortunate development. Data from the Robert Koch Institute (RKI) show a first-dose vaccination rate of about 81% for caretakers in August 2021 [11].

This is a single-facility outbreak investigation; findings cannot be transferred to other settings. Conclusions are drawn from anecdotal evidence and chronologic categorizations. Due to the retrospective study design, further methods, such as sequencing viral strains or cross-sectional testing of entire groups to investigate the chain of infection, could not be applied. It is possible that several asymptomatic infections, especially in children, remained unnoticed. Additionally, despite a comprehensively probing interview technique, we cannot exclude a possible recall bias as a consequence of a time period of two to three months between infection and interview. Although these are limitations of our study, we still believe that it gives relevant insights into infection dynamics in such facilities and that this might help health decision makers to develop effective strategies to avoid outbreaks in the future.

## 5. Conclusions

In this kindergarten case cluster, viral spread seems to have occurred mainly among caretakers, which supports the ongoing endeavor to vaccinate all kindergarten staff. Apart from that, in the light of a currently ongoing fourth epidemic wave in Germany and increasing infection incidence in the youngest age groups as vaccination rates in the above-12-year population increase, strict adherence to hygiene concepts and immediate response algorithms following positive cases seem advisable to avoid future outbreaks in kindergartens.

## Figures and Tables

**Table 1:** Symptoms reported for RT-PCR positive-tested children in a Berlin Kita outbreak, November/December 2020.

Children *	Fever	Cold ^1^	GI ^2^	Exhaustion ^3^	Sense ^4^
C-5 (1)	■	■		■	
C-5 (2)	■	■		■	
C-6	■	■	■		
C-7 (1)	■		■	■	
C-7 (2)		■			
C-11					
C-14	■		■		

* Individual and household number corresponding with Figure 1; C, child. ^1^ Cold, symptoms referring to a common cold, such as a cough, runny nose, headache, and chill; ^2^ GI, gastrointestinal symptoms, such as diarrhea, nausea, emesis, and stomachache; ^3^ Exhaustion, complaints like fatigue, limb pains, and weakness; ^4^ Sense, loss of senses (smelling, taste). ■, respective symptoms reported.

**Table 2:** Symptoms reported for RT-PCR positive-tested adults in a Berlin Kita outbreak, November/December 2020.

Adults *	Fever	Cold ^1^	GI ^2^	Exhaustion ^3^	Sense ^4^
E-1			■	■	■
E-2	■	■	■		■
E-3		■		■	
E-4	■	■		■	
P-4		■			
P-5 (1)	■	■		■	■
P-5 (2)	■	■		■	
P-6	■		■	■	■
P-7 (1)	■	■		■	■
P-7 (2)		■		■	■
E-8		■	■	■	
E-9		■	■		■
E-10	■	■		■	
P-11		■		■	■
E-12		■	■	■	■
E-13		■			■

* Individual and household number corresponding with Figure 1; E, employee; P, parent. ^1^ Cold, symptoms referring to a common cold, such as a cough, runny nose, headache, and chill; ^2^ GI, gastrointestinal symptoms, such as diarrhea, nausea, emesis, and stomachache; ^3^ Exhaustion, complaints like fatigue, limb pains, and weakness; ^4^ Sense, loss of senses (smelling, taste). ■, respective symptoms reported.

## Data Availability

Data are not stored publicly but can be made available upon reasonable request from the corresponding author.
